# Quasi‐Amorphous Metallic Nickel Nanopowder as an Efficient and Durable Electrocatalyst for Alkaline Hydrogen Evolution

**DOI:** 10.1002/advs.201801216

**Published:** 2018-10-20

**Authors:** Doudou Zhang, Jingying Shi, Yu Qi, Xiaomei Wang, Hong Wang, Mingrun Li, Shengzhong Liu, Can Li

**Affiliations:** ^1^ Key Laboratory of Applied Surface and Colloid Chemistry Ministry of Education Shaanxi Key Laboratory for Advanced Energy Devices Shaanxi Engineering Lab for Advanced Energy Technology Institute for Advanced Energy Materials School of Materials Science and Engineering Shaanxi Normal University Xi'an 710119 P. R. China; ^2^ Dalian National Laboratory for Clean Energy *i*ChEM Dalian Institute of Chemical Physics Chinese Academy of Sciences Dalian 116023 China

**Keywords:** alkaline solutions, chemical synthesis, electrocatalysts, hydrogen evolution reaction, quasi‐amorphous metallic nickel

## Abstract

Nickel is regarded as the best alternative metal electrocatalyst to platinum for hydrogen evolution reaction (HER). Success in developing a quasi‐amorphous metallic nickel (QAMN) nanopowder catalyst using a two‐step chemical route for efficient and durable HER in alkaline solution is reported. It is found that the QAMN electrocatalyst exhibits essentially zero overpotential at the cathodic onset while delivering 10 mA cm^−2^ at an overpotential of only 240 mV; both performances are far better than what was reported previously using prior metallic nickel catalysts. Taking into account increased surface area, further enhanced activity is attributed to the superior intrinsic activity. Meanwhile, the QAMN catalyst shows excellent stability in accelerated and interrupted polarization in alkaline solution for tens of hours. This study provides a new chemical means to prepare amorphous metallic materials for more efficient catalysis.

## 1 Introduction

Electrochemical hydrogen generation from water splitting powered by clean solar and wind electricity has been regarded as one of the most promising approaches to store the intermittent renewable energy.^1^, ^2^ Extensive efforts have been made on the alkaline electrolysis since this technology is able to offer high purity hydrogen with great promise for large‐scale production at low cost.^3^ In fact, most of the electrochemical water splitting devices used to generate hydrogen in a few limited commercial applications such as metallurgy, semiconductor processing, glass cutting, acrylic polishing, etc. are based on alkaline electrolysis technology. However, in alkaline solution, the rate for hydrogen evolution reaction (HER) is at least two orders of magnitude slower than that in acidic environment, which is a great drawback for the alkaline electrolysis.^4^, ^5^, ^6^, ^7^ Moreover, it needs to use very noble metal (platinum) HER electrocatalysts to attain low overpotential at the onset and rapid current increase with the voltage applied.^8^, ^9^, ^10^, ^11^ To achieve effective hydrogen generation at affordable cost, it is of crucial importance to develop an effective electrocatalyst using earth‐abundant elements for the hydrogen production in alkaline solution.

On the basis of theoretical studies, Trasatti and Nørskov et al. predicted that metallic nickel is the best alternative to platinum electrocatalyst for HER according to the volcano plot which demonstrates the relationship between the exchange current density and the calculated hydrogen adsorption energies.^12^, ^13^, ^14^ There have been some experimental evidences to confirm the theory that the metallic nickel is indeed more active than other nonnoble metals for the HER electrocatalysis.^15^, ^16^, ^17^ Meanwhile, quite a few Ni‐based alloys and compounds with various heterostructures have been developed to demonstrate much improved electrocatalytic activities over pristine metallic nickel nanocrystals, as summarized in recent review articles.^18^, ^19^

In comparison with a well‐crystallized nanomaterial, its amorphous counterpart often supplies abundant defect sites as active centers for enhanced catalytic performance, especially for the electrocatalytic process that generally requires redox centers to facilitate transportation of charged species. For example, an amorphous cobalt phosphate catalyst prepared by in situ electrodeposition achieves considerable high activity for electrochemical oxygen evolution in neutral electrolyte.^20^ Likewise, the electrocatalytic HER activity of MoS_2_ in amorphous phase was intrinsically enhanced due to more active sites of the unsaturated sulfur atoms and higher surface area in contrast to its crystalline phase.^21^, ^22^, ^23^, ^24^, ^25^, ^26^, ^27^ Although various methods such as electrodeposition, thermal plasma, polyol process, chemical vapour deposition, chemical reduction in the liquid phase have been developed for synthesizing metallic nickel powders or thin films, they all evidently demonstrate crystalline characteristics despite their nanoscale sizes even in isolated single‐atom state.^28^, ^29^, ^30^, ^31^

Herein, we report a two‐step chemical route to synthesize quasi‐amorphous metallic nickel (QAMN) nanopowder under the presence of Mo and W related impurity species. The QAMN catalyst demonstrates much higher electrocatalytic HER activity than its crystallized metallic nickel (CMN) counterpart prepared by the same method, which significantly outperforms the previously reported metallic nickel catalysts.^28^, ^29^, ^30^, ^31^, ^32^, ^33^, ^34^ Specifically, the overpotential required for the QAMN catalyst to generate cathodic current density of 10 mA cm^−2^ is only 240 mV, which is 162 mV lower than its crystalline reference synthesized using the same chemical route. Notably, the QAMN catalyst has an onset potential close to zero overpotential. To our knowledge, this is exceptional for a nonnoble electrocatalyst.^35^, ^36^ Besides, it demonstrates remarkable robustness against the continuous and interval long‐term polarization in alkaline electrolysis. A mechanism for synthesis of the QAMN powder is provided with its outstanding HER activity discussed.

## 2 Results and Discussion

**Figure 1:**
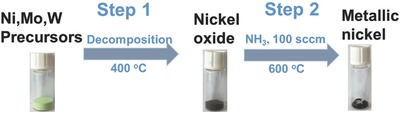
Schematic illustration of the two‐step method for preparation of metallic nickel nanopowders. NiCO_3_ · 2Ni(OH)_2_ · 4H_2_O was used as bulk raw material, while (NH_4_)_6_Mo_7_O_24_ and (NH_4_)_6_H_2_W_12_O_40_ · XH_2_O as doping raw materials for synthesizing Ni, Mo, W precursors by ion exchanging.

The metallic nickel nanopowder was synthesized by a two‐step chemical route as schematically illustrated in **Figure** **1**. The (Mo, W)‐doped hydrotalcite‐like basic nickel carbonate was prepared by ion exchanging for precursor materials. The feeding nickel, molybdenum, and tungsten in atomic ratio were set at 1:0.4:*x* (*x* = 0, 0.05, 0.07, 0.1, 1.0). In the first, the powder‐like oyster yellow precursors were heated at 400 °C in air for 2 h to generate powders in brown. Secondly, the brown powders were further annealed at 600 °C under a continuous flow of ammonia gas and the black powders were collected for characterization and fabrication of electrocatalyst.

The crystal phase of the intermediates (derived at the first step) and the final products were checked by XRD analysis. As seen in Figure S1 (Supporting Information), the precursors were fully resolved and the NiO phase (JCPDS card #47‐1049) appeared at the first process while it converted into metallic Ni (JCPDS card #04‐0850) after the second process. Compared with the samples prepared under *x* = 0, the diffraction peaks of the NiO and Ni for those samples obtained at *x* = 0.05–1 were broader with lower intensities, and even disappeared in the case of nickel metal derived at *x* = 0.07 under the same intensity scale. Remarkably, the achieved crystallinity of the intermediated NiO and final Ni is strongly related to the amount of tungsten additive in the precursors. Based on the XRD results, we defined the final samples prepared at *x* = 0 as CMN and *x* = 0.07 as QAMN for studying the crystallinity effect. Meanwhile, the intermediate sample derived in the first stage before it was converted into the CMN or QAMN was denoted as the corresponding B‐CMN or B‐QAMN (B stands for before) herein after for discussion.

**Figure 2:**
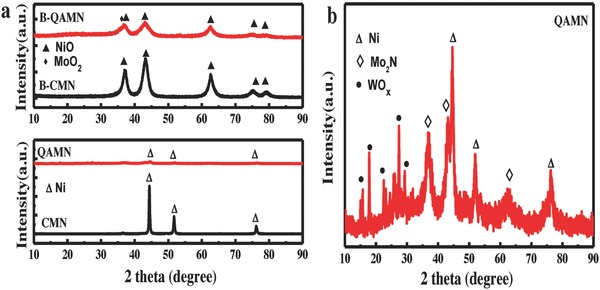
a) XRD patterns of the CMN, QAMN, B‐CMN, and B‐QAMN samples. b) Enlarged XRD pattern of the sample QAMN.

**Figure** **2**a summarizes the powder XRD patterns of the CMN, QAMN, B‐CMN, and B‐QAMN samples. A separated MoO_2_ phase (JCPDS card #50‐0739) is seen in the sample B‐QAMN, while Mo_2_N (JCPDS card #25‐1366) and WO*_x_* (mixed phases of WO_3_ and WO_2_, JCPDS cards #20‐1323, #32‐1393, 2θ range of 20°–30°) can be discerned from the enlarged pattern of the sample QAMN shown in Figure 2b. However, no XRD signals related to impurity phases can be clearly found in the samples of B‐CMN and CMN, which may be conceived by the intense diffraction of the NiO or Ni crystals. In this respect, a high doping level up to 40% of Mo does not show perceptible effect on the crystal growth of the dominating phases of NiO and Ni. Howbeit, when the extra W species with low amount of 7% were added as codopant, the crystallization of NiO was significantly deteriorated and the poor CMN was acieved as a result. The tungsten precursor species (H_2_W_12_O_40_^6−^) has much larger size than the raw molybdenum species (Mo_7_O_24_^6−^), which may account for the above different behaviors. In addition, the nickel crystal unit has much smaller volume than that of the nickel oxide (both cubic units with the side length of 3.5238 Å for metal and 4.1771 Å for oxide). For this reason, the crystallization of metallic nickel may be more susceptible to the implantation of foreign species than its oxide phase. As seen in Figure S1 (Supporting Information), the NiO with low crystallinity in the first stage is requisite to give metallic nickel with further distorted crystal structure in the second stage. Therefore, when the poor crystallized NiO is reduced to be metallic nickel with smaller cell volume, the structure distortion is further aggravated and finally results in the formation of quasi‐amorphous nickel phase.

Furthermore, we synthesized the target product with the feeding ratio of Ni:Mo:W as 1:0:1. Note that the tungsten ratio is sufficiently high and there is no molybdenum used in this case. The products derived at the first and second steps are designated as B‐Ni_W_ and Ni_W_ for discussion, respectively. It is found that without molybdenum (with only tungsten species), a similar phase evolution during synthesis can be observed from the XRD patterns in Figure S1c,d (Supporting Information), NiO for the B‐Ni_W_ and metallic nickel for the Ni_W_ in addition to WO*_x_* phase. The XRD patterns for the B‐CMN, B‐QAMN, CMN, and QAMN samples are also listed for comparison. It is seen that the sample Ni_w_ demonstrates lower diffraction intensity for metallic Ni than the sample CMN, yet remarkably higher than the sample QAMN. Similar result is found for NiO phase in the sample B‐Ni_w_, which exhibits weaker diffraction peaks than the B‐CMN but stronger than B‐QAMN. These facts reveal that the metallic nickel with extremely low crystallinity is hard to achieve even under the presence of large amount of tungsten species for synthesis. As already indicated above, in case of Mo species (without W) for synthesis, both Ni and NiO can be well crystallized (the sample CMN for Ni and the sample B‐CMN for NiO). Therefore, Mo and W together are very crucial for the successful synthesis of metallic nickel with extremely low crystallinity by the present two‐step method.

**Figure 3:**
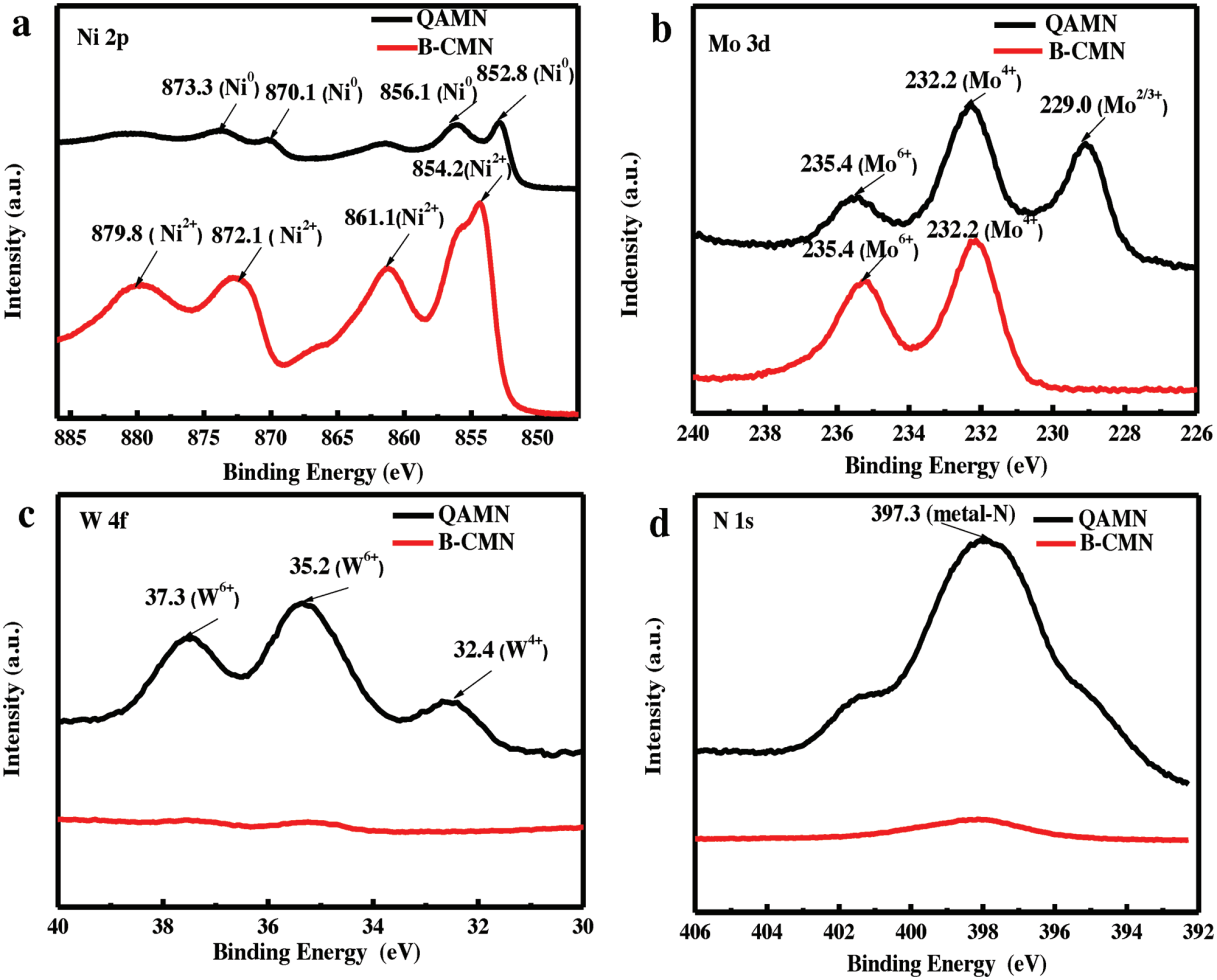
XPS spectra of the B‐CMN and QAMN samples. a) Ni 2p. b)Mo 3d. c) W 4f. d) N 1s.

To identify elements and their chemical states, we used the X‐ray photoelectron spectroscopy (XPS) technique to detect and the sample QAMN with B‐CMN for comparison. The wide‐scan spectra (Figure S2, Supporting Information) show the presence of Ni, Mo, W, O, and N elements for the former, and Ni, Mo, and O elements for the latter, which reveals the nitridation after NH_3_ annealing. **Figure** **3** demonstrates the separate XPS spectra for Ni 2p, Mo 3d, W 4f, and N 1s in the above two samples. In Figure 3a, the two main peaks for Ni 2p at 854.2 and 872.1 eV for its 2p3/2 and 2p1/2 levels with their corresponding satellite peaks on the high energy side can be assigned to Ni^2+^ species from nickel oxide, while those characteristic and satellite peaks at 852.8, 856.1 eV and 870.1, 873.3 eV can be attributed to metallic nickel with Ni^0^ chemical state.^34^ There are no other valences for nickel that could be identified. It thus confirms the thorough conversion of nickel oxide into metallic nickel rather than nitrides those are generally derived by annealing in ammonia gas, which agrees well with the above XRD results. In the Mo 3d spectra (Figure 3b), the peaks located at 229.0 and 232.2 are indexed to the Mo^2/3+^ species in Mo_2_N and the state of Mo^4+^ in MoO_2_.^37^, ^38^, ^39^ The peak at higher binding energy of 235.4 eV is defined to the oxidative state of Mo^6+^ in MoO_3_, which is often presented together with the metastable MoO_2_ due to its surface oxidation in air.^36^ The signals of Mo^4+^ and Mo^6+^ are both observed in the two samples while the Mo^2/3+^ state only appears in the NH_3_‐treated sample. From this result, it is seen that the molybdenum oxides were partially converted into nitride during annealing in ammonia gas. In Figure 3c, no XPS signals of tungsten could be found for the sample B‐CMN that prepared without additive of tungsten species. For the sample QAMN, the two characteristic peaks at 37.3 and 35.2 eV could be ascribed to the W^6+^ state in WO_3_, while the peak at the low binding energy of 32.4 eV was assigned to WO_2_ with W^4+^ state.^40^ This result is well verified by using a single ammonium metatungstate as precursor for the same two‐step process, which generates WO_3_ firstly and the mixed phases of WO_3_ and WO_2_ finally (XRD patterns in Figure S3, Supporting Information). In the N1s spectra (Figure 3d), the peak at 397.3 eV is assigned to N‐Mo species for the sample QAMN while no signal for N is found for the sample B‐CMN without NH_3_ treatment.^40^ Thus, by treating under NH_3_ flow at 600 °C, Ni^2+^ species are entirely reduced to be Ni^0^ state; in contrast, the reduction reactions of molybdenum and tungsten species with high valence state are far incomplete. Besides, only molybdenum oxide can be nitridized to build metal‐nitrogen bond under the current conditions.

Raman spectroscopy was also used to identify the emerging metallic phase, as seen in Figure S4 (Supporting Information). Two strong bands at 500 and 850 cm^−1^ are detected for the sample B‐QAMN while no signal is found for the sample QAMN over the wavenumber range of 100–1800 cm^−1^. The absence of Raman characteristic band over 100 cm^−1^ is commonly considered as signature of metallic phase, and thus it confirms the phase transition and the final metallic phase. Electroconductivity and magnetic properties are also tested for confirmation of the phase change before and after NH_3_ treatment. The as‐prepared powders were compressed into tablets for electroconductivity test. The square resistance is as high as ΜΩ scale, but it sharply decreases to mΩ level after nitridation, indicating the conversion from insulator into conductor. The B‐QAMN powder shows no response to a permanent magnet, while the QAMN powder is strongly allured to the magnet surface, as seen in the digital pictures shown in Figure S5 (Supporting Information). The commercial NiO powder is likewise nonmagnetic, which turns to be magnetic after annealing under NH_3_. Thus, it is apparent that under the present condition using ammonia as reducing agent, the nickel oxide is selectively converted into metallic nickel rather than its nitride phase.

According to the XPS analysis, the surface atomic ratio of Ni:Mo:W of the sample QAMN was evaluated to be 97:2:1. This result is very close to the bulk composition that determined by the inductively coupled plasma atomic emission spectrometry the ratio of Ni:Mo:W in weight as 85.7:2.7:2.7, i.e., 100:2:1 in atomic ratio. So, nickel remarkably dominates the catalyst while the Mo and W species are the actual impurity with low contents roughly as 2% and 1%, respectively. Likewise, metallic nickel also is the dominating component of the sample CMN.

**Figure 4:**
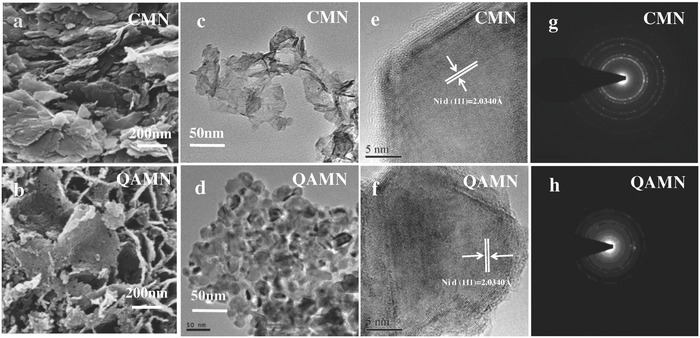
a,b) SEM images; c,d) TEM images; e,f) HRTEM images; g,h) SAED patterns of the CMN and QAMN powder samples.

The morphological properties of the samples CMN and QAMN are illustrated and compared in **Figure** **4**a–d. It is seen from the scanning electron microscopy (SEM) images (Figure 4a,b) that both the samples exhibit the layered sheet‐like morphology possibly inheriting from the hydrotalcite‐like nickel carbonate precursor. Compared with dense and smooth nanosheets for the CMN powder, the rough surface with individual particles about 25 nm in size can be observed for the QAMN nanosheets. The transmission electron microscopy (TEM) images (Figure 4c,d) confirm the above results. As expected, the sample QAMN has a significantly higher surface area of 147.3 m^2^ g^−1^ as determined by the Brunauer–Emmett–Teller technique (Figure S6, Supporting Information), which is about eight times higher than that of CMN. The crystalline state can be learned from the high‐resolution transmission electron microscopy (HRTEM) imaging (Figure 4e,f) and the selected area electron diffraction (SAED) patterns (Figure 4g,h). Clear and orderly lattice fringes with separate diffraction rings are observed for CMN powder, suggesting well crystallized and polycrystalline in nature. In contrast, the sample QAMN demonstrates fuzzy and random lattice fringes with broad halos and dim rings in SAED pattern, the crystallinity of which is so poor that it may be defined to be quasi‐amorphous state as revealed by the XRD results.

To evaluate electrochemical activity, the as‐prepared nanopowders were transferred onto the glassy carbon electrode (GCE) by using Nafion binder agent. The as‐fabricated catalysts were tested as working electrode in Ar‐saturated 0.1 m KOH electrolyte for HER using a standard three‐electrode setup. For fair comparison, the mass loading of catalyst was kept at 0.26 ± 0.02 mg cm^−2^. The electrochemical HER activity is dominated by the metallic Ni rather than the impurity phases of Mo_2_N and WO*_x_*. Firstly, tungsten oxides are generally inactive toward alkaline HER,^41^ not to mention that in the present case, the amount of WO*_x_* is very small. As for the Mo_2_N, although there have been reports on its activity as a HER electrocatalyst,^42^, ^43^ it is not as dominating as QAMN. In fact, we measured the linear sweep voltammetry (LSV) curves of the as‐prepared catalysts. As shown in Figure S7 (Supporting Information), the QAMN sample displays much higher electrocatalytic activity than other samples using various W ratios with *x* = 0, 0.05, 0.1 in the precursors. Considering that the constant ratio of Ni to Mo was used for synthesis, these catalysts should contain comparable amount of metallic nickel and Mo_2_N. In view of the big difference in activity, it is believed that the HER activity is dominated by the QAMN. Meanwhile, the sample Ni_W_ demonstrates low HER activity (Figure S7, Supporting Information), confirming that the metallic nickel plays a dominant role towards the HER.

**Figure 5:**
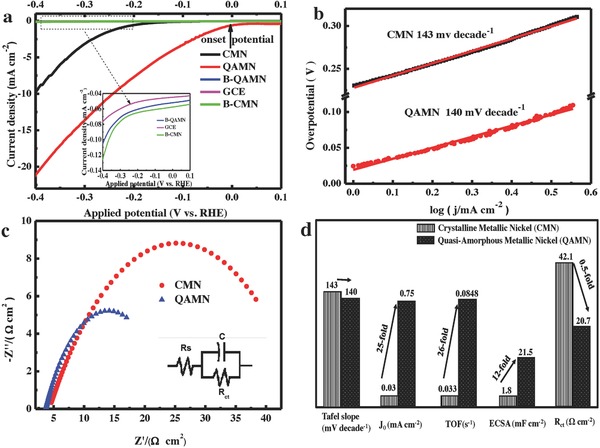
Electrocatalytic HER activity and durability in 0.1 m KOH solution. a) LSV curves with scan rate of 5 mV s^−1^. The inset shows the details for the GCE, B‐CMN, and B‐QAMN electrodes. b) Tafel plots of the CMN, QAMN electrodes derived from their LSV curves. c) Nyquist plots of the CMN and QAMN electrodes at an overpotential of 0.1 V. The inset is the equivalent circuit used for fitting. d) The determined parameters of the QAMN and CMN electrocatalysts for alkaline HER.

In order to correlate HER activity with crystallinity, the sample QAMN and the sample CMN were characterized by more electrochemical measurements, and the results were fully compared and discussed for more insight. The LSV curves of the above two electrodes together with other referenced catalysts are presented in **Figure** **5**a. It is found that the nickel oxide catalysts, either from sample B‐CMN or B‐QAMN, are as inert as the GCE substrate, which agrees well with the previous reports.^44^, ^45^, ^46^ By contrast, the metallic nickel catalysts of both QAMN and CMN phases are remarkably active toward the alkaline HER. Furthermore, the QAMN catalyst demonstrates the highest activity with overpotential as low as 240 mV for it to generate a benchmark current density of 10 mA cm^−2^, performing much better than the previously reported metallic nickel catalysts (see details in Table S1, Supporting Information). However, at least 162 mV of excess bias has to be applied for the CMN catalyst to deliver the same current density for alkaline HER. Notably, the QAMN has an onset potential approaching 0 mV, which is seldom achieved by the nonnoble metal catalysts up to present.^47^, ^48^, ^49^, ^50^, ^51^, ^52^

Figure 5b compares the Tafel plots of the CMN and QAMN catalysts derived from LSV curves. The curves are fitted to the Tafel equation η = *a* + *b* log (*j*), where *j* is the current density and *b* the Tafel slope. The Tafel slopes are 140, 143 mV dec^−1^ for the QAMN and CMN catalysts, respectively, indicating the Volmer–Heyrovsky mechanism.^42^ The exchange current density for the QAMN is estimated to be 0.75 mA cm^−2^, about 25‐fold of that for the CMN (0.03 mA cm^−2^). As such, the long distance disorder in the crystal structure does not change the reaction mechanism, but it significantly boosts the reaction rate.

On a per‐surface‐atom basis, the measurement of mass‐specific catalytic activities enables estimation to achieve more insight given a series of approximations regarding particles composition and morphology.^15^ Figure S8 (Supporting Information) shows the mass loading versus current density measured at a constant overpotential of 200 mV for the CMN and QAMN electrodes. Both curves are apparently fitted well using the simple power law equation, with parameters summarized in Table S2 (Supporting Information). Assuming that the nanoparticles are uniformly distributed throughout the material, the amount of surface atoms can be estimated. Therefore, the turnover frequencies (TOFs) are calculated to be 0.033 and 0.848 s^−1^ for the CMN and QAMN powder catalysts, respectively (details in Supporting Information). In other words, the QAMN catalyst exhibits 26 times as active as the CMN for the HER. So, the lattice disorder may offer more active sites for the interfacial reaction.

As the electrochemical surface area (ECSA) is another important factor for the enhanced activity, we measured the electrochemical capacitance of the film–electrolyte interface in double‐layer regime of voltammograms to determine the relative ECSA for the QAMN and CMN electrodes.^46^, ^47^, ^52^ The positive and negative capacitance currents at the centers of the potential window are averaged and plotted against the scan rates, as shown in Figure S9 (Supporting Information). Assuming that the intrinsic‐specific surface capacitance is approximately the same for all films, ECSA of the quasi‐amorphous phase is about 12‐fold larger than that of the crystalline catalyst (21.5 vs 1.8 mF cm^−2^ for double‐layer capacitance). In view of the higher folds of 25 and 26, respectively, for the increase in the exchange current density and TOF than that of the ECSA with 12‐fold enhancement, it clearly reveals an intrinsic improvement in HER activity when the nickel crystalline catalyst is collapsed into quasi‐amorphous structure.

To gain further insight into the catalytic mechanism, we conducted electrochemical impedance spectroscopy (EIS) at an overpotential of 200 mV and compared the Nyquist plots for the QAMN and CMN catalysts. Figure 5c shows that the interfacial charge transfer resistance (*R*_ct_) for the QAMN is about half of that for the CMN catalyst (20.7 vs 42.1 Ω cm^2^), suggesting a superior alkaline HER kinetics over the quasi‐amorphous catalyst. In this regard, the boosted activity should not be attributed to high electric conductivity of the metallic catalyst. So, the decreasing crystallinity actually contributed to the fast charge transfer at the solid–electrolyte interface.

The above parameters derived for the CMN and the QAMN catalysts are sumarized and compared in Figure 5d. The similar Tafel behavior and different *R*_ct_ indicate that the QAMN and CMN catalysts follow the same HER route and the conductivity is not the reason for the changed electrochemical activity. In addition, the QAMN exhibits 25‐fold in exchange current density, 26‐fold in TOF, 12‐fold in ECSA as large as those of the CMN. It is seen that the increasing rates of the intrinsic parameters of *j*_0_ and TOF are over double that of ECSA. These fundamental data reveal that the improved HER activity of QAMN is originated from the intrinsic properties more than the single geometric factor of ECSA.

**Figure 6:**
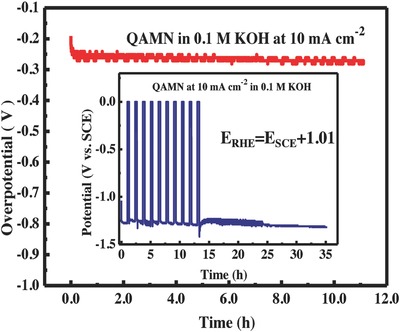
Time dependence of overpotential for the QAMN electrode recorded at 10 mA cm^−2^ in Ar‐saturated 0.1 m KOH solution at room temperature. The inset is cyclic current interruption test with following continuous polarization. (*E*_RHE_ (V) = *E*_SCE_ (V) + 1.01(V)).

The electrochemical stability is another key figure‐of‐merit for the electrocatalysts. We measured the chronoamperometry curve using the QAMN electrode at the current density of 10 mA cm^−2^ for 10 h. **Figure** **6** indicates that the overpotential remains unchangeable and Faraday efficiency is close to 100% in the testing duration (Figure S10, Supporting Information). Furthermore, cyclic current interruption was used to test the QAMN electrode for its tolerance against the power on/off and alkaline soaking (the inset in Figure 6). Apparently, outstanding chemical stability is demonstrated by keeping constant overpotential regardless of current interruptions during the polarization test of 36 h.

## 3 Conclusion

In conclusion, we successfully developed a chemical route for the systhesis of quasi‐amorphous nickel metal catalysts, which exhibits superior alkaline HER activity with cathodic onset potential approaching 0 mV and overpotential required as low as 240 mV to deliver current density of 10 mA cm^−2^. The QAMN also demonstrates the robust performance in sustaining or intermittent polarization for tens of hours. In addition to the increasing ECSA, the enhancement in the intrinsic activity is the primary reason for the outstanding HER performance of the QAMN compared with its crystalline phase catalyst. This study affords a new strategy to prepare QAMN catalysts with great promise for efficient water electrolysis.

## Conflict of Interest

The authors declare no conflict of interest.

## Supporting information

Supplementary
