# Supplementary material for: Quasi‐Amorphous Metallic Nickel Nanopowder as an Efficient and Durable Electrocatalyst for Alkaline Hydrogen Evolution
Source: Adv Sci (Weinh). 2018 Oct 20;5(12):1801216. doi: 10.1002/advs.201801216 (PMC6299734; doi:10.1002/advs.201801216)
Supplement: Supplementary file 1 — Supplementary [file ADVS-5-1801216-s001.pdf]

Copyright WILEY-VCH Verlag GmbH & Co. KGaA, 69469 Weinheim, Germany, 2018.

ADVANCED SCIENCE
Open Access

Supporting Information

for *Adv. Sci.,* DOI: 10.1002/advs.201801216

Quasi-Amorphous Metallic Nickel Nanopowder as an Efficient and Durable Electrocatalyst for Alkaline Hydrogen Evolution

Doudou Zhang, Jingying Shi, Yu Qi, Xiaomei Wang, Hong Wang, Mingrun Li, Shengzhong Liu,* and Can Li*

Supporting Information

Title: Quasi-Amorphous Metallic Nickel Nanopowder as Efficient and Durable Electrocatalyst for Alkaline Hydrogen Evolution

Doudou Zhang, Jingying Shi, Yu Qi, Xiaomei Wang, Hong Wang, Mingrun Li, Shengzhong Liu, Can Li

D. D. Zhang, Prof. S. Z. Liu
Key Laboratory of Applied Surface and Colloid Chemistry, Ministry of Education; Shaanxi Key Laboratory for Advanced Energy Devices; Shaanxi Engineering Lab for Advanced Energy Technology; Institute for Advanced Energy Materials; School of Materials Science and Engineering, Shaanxi Normal University, Xi'an 710119, P. R. China.
E-mail: szliu@dicp.ac.cn

D. D. Zhang, Prof. J. Y. Shi, Dr. Y. Qi, X. M. Wang, H. Wang, Prof. M. R. Li, Prof. S. Z. Liu, Prof. C. Li
Dalian National Laboratory for Clean Energy; iChEM, Dalian Institute of Chemical Physics, Chinese Academy of Sciences, Dalian, 116023, China.
E-mail: jingyingshi@dicp.ac.cn; szliu@dicp.ac.cn

Keywords: Quasi-Amorphous Metallic Nickel, Electrocatalyst, Synthesis, Hydrogen Evolution reaction, Alkaline solution

Corresponding authors email. jingyingshi@dicp.ac.cn; szliu@dicp.ac.cn

Experimental Section

1. Precursors prepared by ion exchanging route.

Basic nickel carbonate ($NiCO_3 \cdot 2Ni(OH)_2 \cdot 4H_2O$) (6.18 g), molybdate ($(NH_4)_6Mo_7O_{24} \cdot 4H_2O$) (0.19 g) and metatungstate ($(NH_4)_6H_2W_{12}O_{40} \cdot XH_2O$) (0-14.78 g) were added orderly to 175 mL of mixture solvents that composed of deionized water, triton X-100, and glycol at the volume ratio of 0.66:0.17:0.17. The mixed suspensions were kept stirring for 20 h at 150 ^oC in oil bath using reflux condenser, followed by washing the resultant solid precipitate centrifugation at 5000 rpm and dried at 120 ^oC in air. The as-obtained oyster yellow powders were used as precursor for preparation of metallic nickel by two-step method. All chemicals were analytical grade and were used as purchased without further purification. Solutions were prepared using high purity water (Millipore Milli-Q purification system, resistivity > 18 MΩ cm).

2. Synthesis of metallic nickel nanopowders by the two-step method.

Firstly, the oyster yellow precursor powders were placed in a ceramic crucible and calcinated in the furnace at 400 ^oC for 4 h in air atmosphere. The target temperature was obtained at a rate of 5 °C min^{-1} before it cooled to the room temperature naturally. Secondly, the as-prepared brown powders were transferred to ceramic boat for further annealing in a tube furnace at a higher temperature of 600 ^oC for 90 min under NH_3 atmosphere with flow rate of 100 sccm. The target temperature was likewise obtained at a rate of 5 ^oC min^{-1} and the NH_3 atmosphere was kept constant before cooling to below 30 ^oC in order to prevent the products from carbonizing in air. The resultant black powders were collected and used for characterization and electrode preparation.

3. Characterization for powders.

The structure of the synthesized samples were studied by X-ray Diffraction (XRD) on a Rigaku D/Max-2500/PC powder diffraction meter using Cu Kα radiation (operating voltage: 40 kV, operating current: 200 mA, scan rate: 5° min^{-1}, step size 0.02° in the 2θ range of 10-90°). The morphology of the powder samples was examined by scanning electron microscopy (SEM) using a Quanta 200FEG scanning electron microscope. Transmission electron microscopy (TEM) images and high resolution TEM (HRTEM) were obtained on a Tecnai G^2F30S-Twin (FEI Company) with an acceleration voltage of 300 kV. X-ray Photoelectron Spectroscopy (XPS) was recorded on a Thermo Esca lab 250 equipped with a monochromatic Al Kα X-ray source. The spectra were analyzed using Casa XPS (Casa Software, Ltd.). A standard Shirley baseline without any offset was used for background correction. The C 1s spectrum for adventitious carbon (284.8 eV) was used for charge correction. Raman spectra were recorded on a Renishaw in Via Raman microscope. Single-frequency laser (532 nm, DPSS 532 Model 200) was used as the exciting source for the visible Raman with an output of 2-10 mW and a He-Cd laser (325 nm) was used as the exciting source for the UV Raman. The Ni, Mo, and W contents in final products were measured by inductively coupled plasma atomic emission spectroscopy. The electric resistance of tablet samples were measured by Four-point technology on RTS-9.

4. Fabrication of electrodes from nanopowder catalysts.

The powder catalysts of 4 mg were dispersed in 80 μL Nafion (5 %) solution diluted with 1 mL mixed solvent (deionized water and alcohol at ratio of 4:1) under supersonic stirring to produce viscous suspension. The suspension (5 μL) was then transfer onto the glassy carbon electrode (area: 0.0706 cm^2) with mass loading of 0.26 ± 0.02 mg cm^{-2} and dried under vacuum to be used as working electrode for electrochemical measurements.

5. Electrochemical measurements.

Current density vs. potential curves were recorded with a commercial potentiostat (CHI760D electrochemical workstation) and potential programmer, using a standard three-electrode system, with a Luggin capillary joining the reference electrode (RE) to the working electrode (WE) compartment, and a porous glass frit separating the working electrode and counter electrode (CE) compartments. A graphite rod and a saturated calomel electrode (SCE) were used as the CE and RE, respectively. The presented current density refers to the geometric surface area of the glassy carbon electrode. Electrochemical measurements were carried out in 0.1 M KOH aqueous solution. Applied potential was swept from -0.6 to 0.2 V vs. reversible hydrogen electrode (RHE) with a scanning rate of 5 mV s^{-1}. In all experiments, analytical grade chemicals were used. Water used was obtained by purifying deionized water with a Milli-Q water purification system. All electrochemical experiments are carried out at room temperature (25 ^{o}C).

The faradaic efficiency (FE) of HER catalyzed by QAMN was measured in an H-type electrochemical reactor with a nafion separator. The sample line of the reactor

was connected to online gas chromatography (Agilent 7890 GC, TCD, Argon carrier) to real-time detect the generated hydrogen. Before measurement, the reaction system was thoroughly degassed with ultrapure argon to expel the air. Then, constant current of −10 mA was applied to the electrode and the concentration of hydrogen was analysed with gas chromatography. Calibration was carried out using a similar setup but with two cleaned Pt foils as working and counter electrode, respectively.

Supplementary Figures and Tables

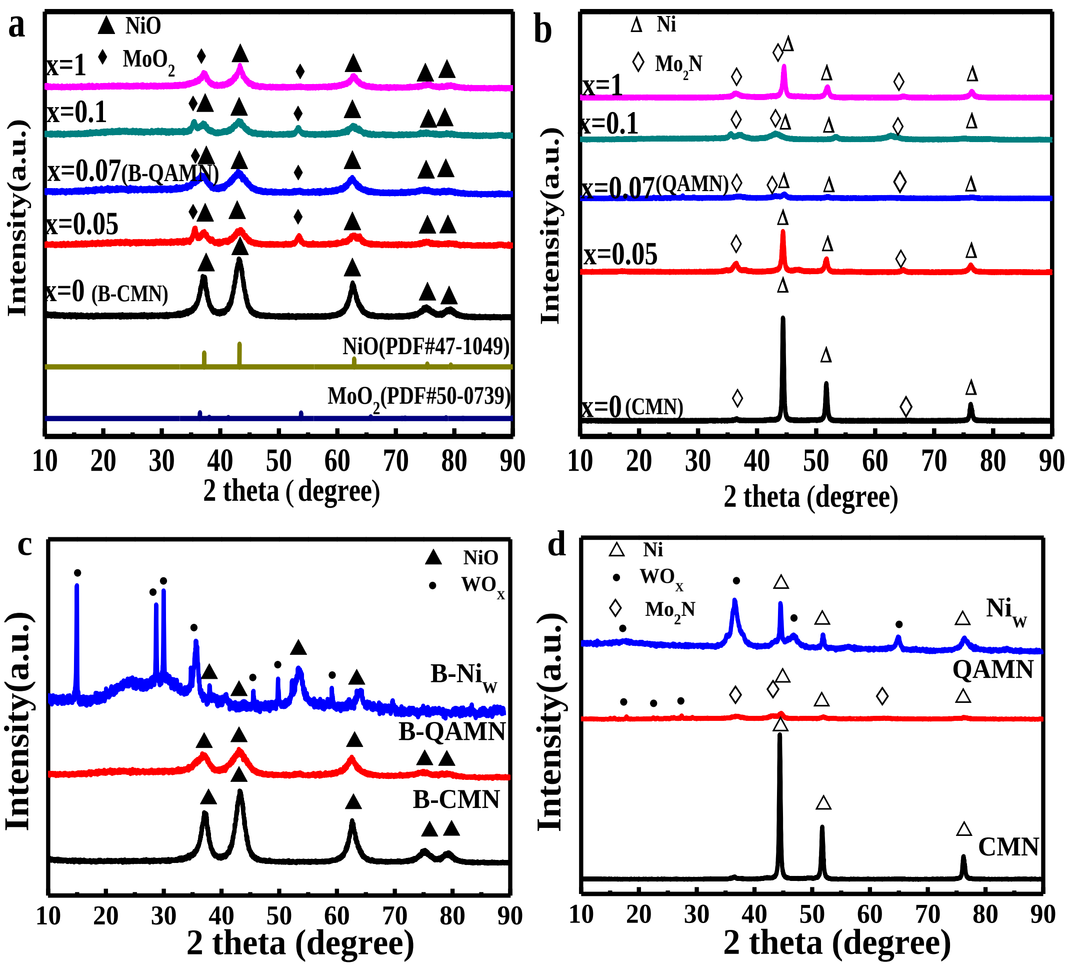

Figure S1. XRD patterns of the samples that obtained at (a) the first step and (b) the second step with various W ratios (x = 0, 0.05, 0.07, 0.1, 1) in precursors. XRD patterns of the B-Ni_W (c) and Ni_W (d) samples with other patterns listed for comparison.

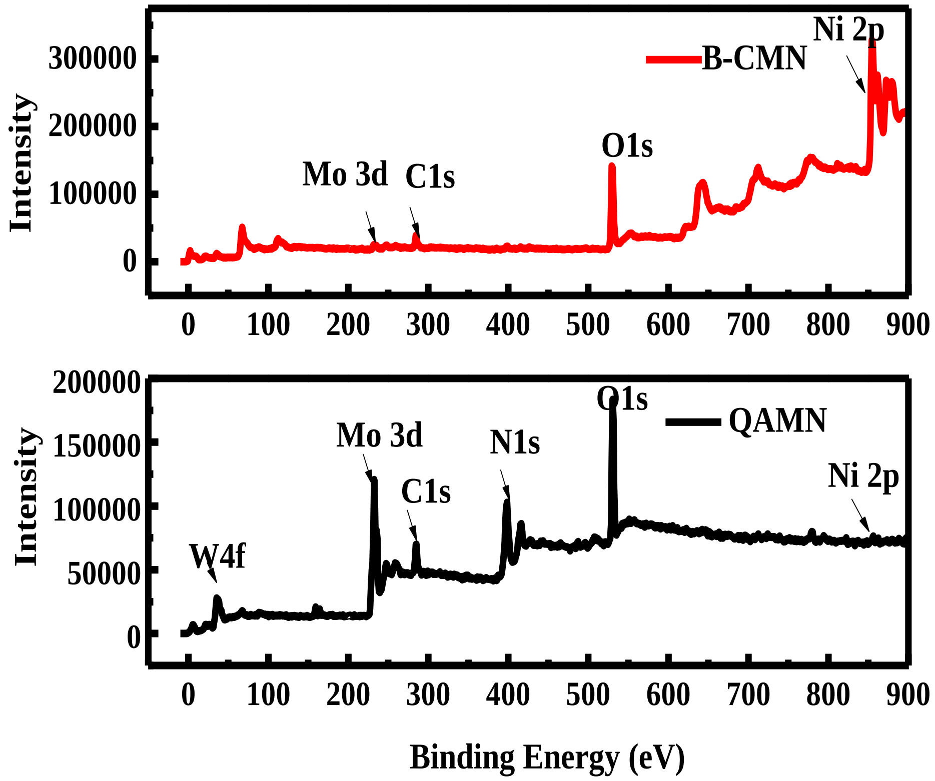

Figure S2. Wide-scan XPS spectra of the B-CMN and QAMN powder samples.

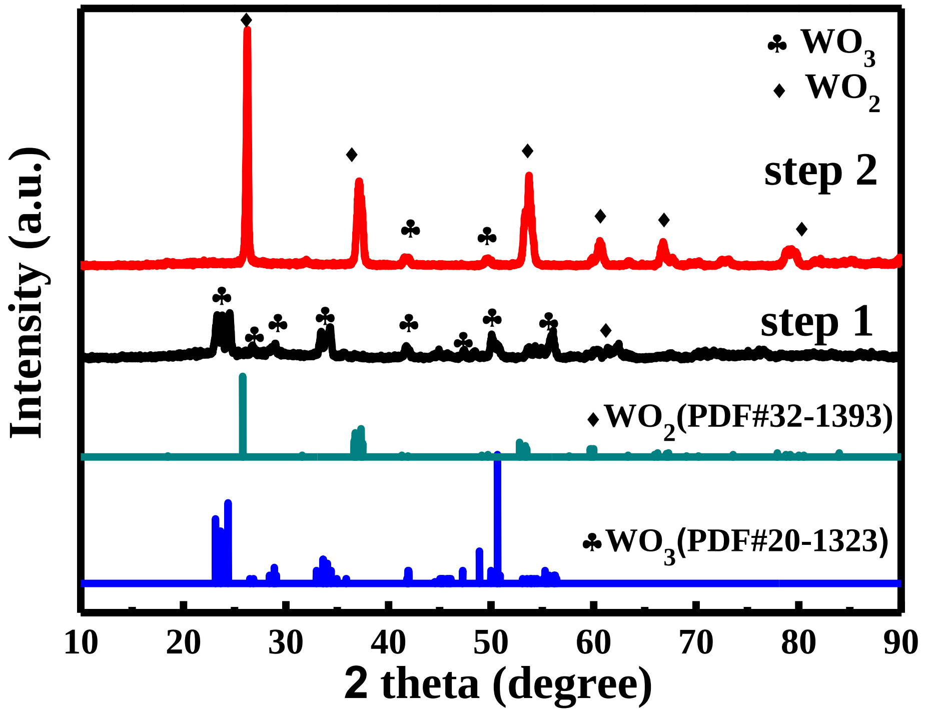

Figure S3. XRD patterns of the powder products obtained at step 1 and step 2 using $(NH_4)_6H_2W_{12}O_{40}\cdot XH_2O$ as single precursor (step 1: air treatment at 400 ^{o}C for 4 hours; step 2: NH_3-treatment at 600 ^{o}C for 1.5 hours).

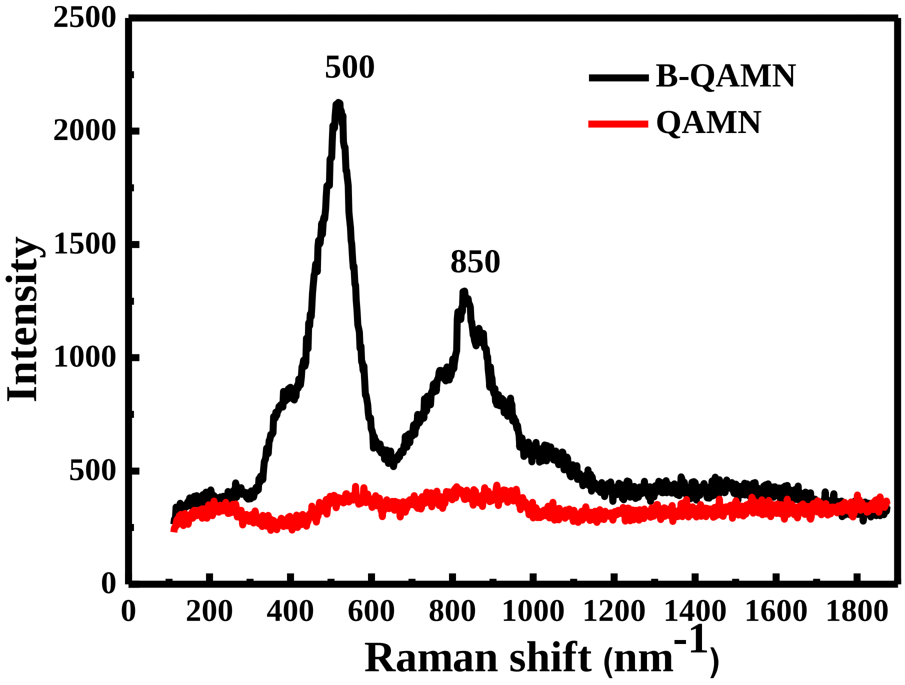

Figure S4. Raman spectra of the B-QAMN and QAMN powder samples.

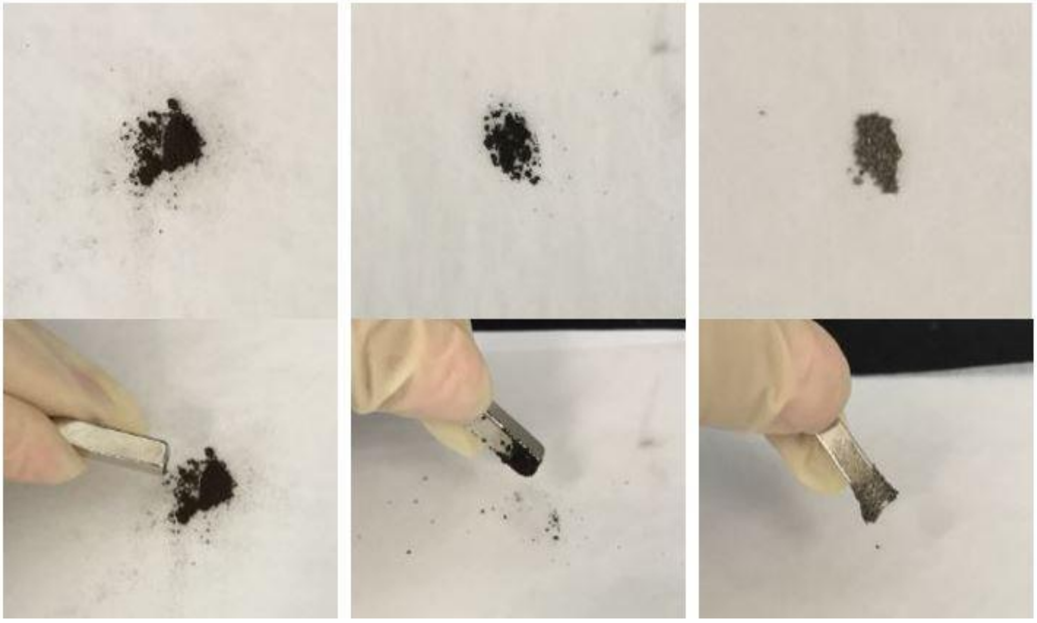

Figure S5. The digital pictures for magnetic testing.

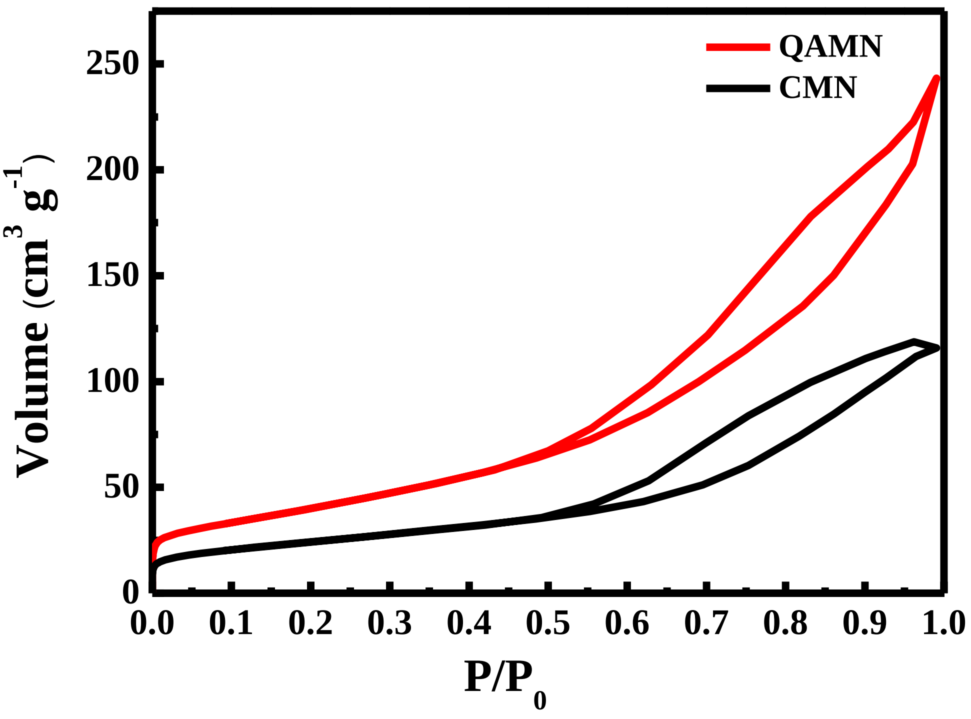

Figure S6. N_2 adsorption-desorption isotherm of the sample CMN in contrast to the sample QAMN.

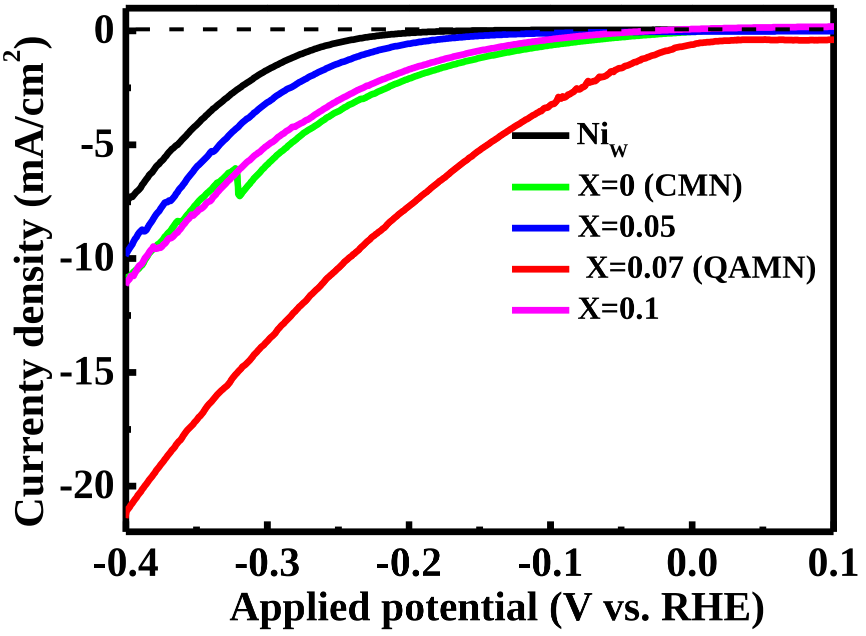

Figure S7. LSV curves of the metallic nickel catalysts prepared with the various W ratios (x = 0, 0.05, 0.07, 0.1) and Ni_W (Ni:Mo:W=1:0:1 for precusor synthesis) in precursor solutions. Electrolyte: 0.1 M KOH solution; Scan rate: 5 mV s^{-1}.

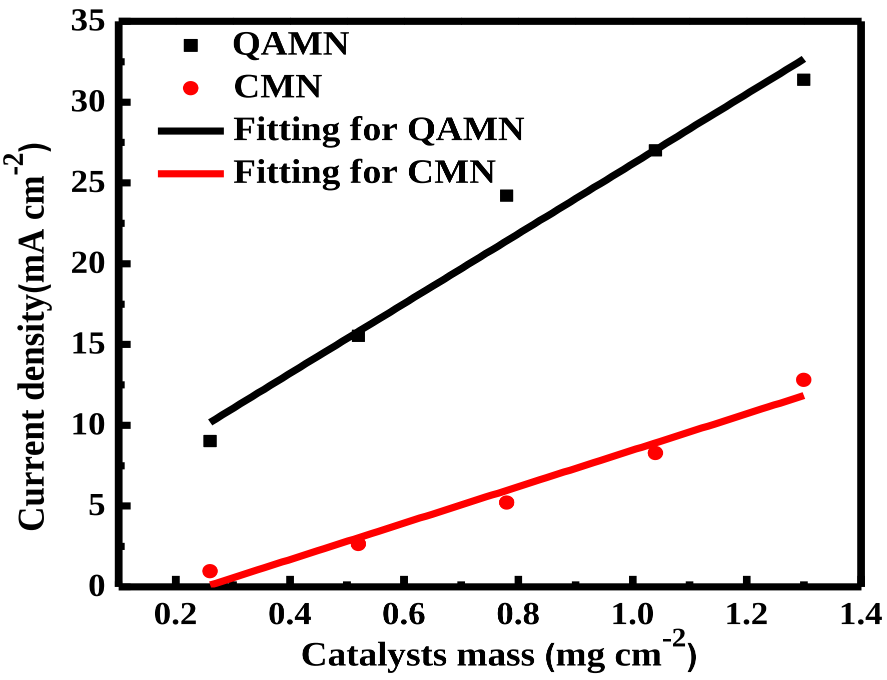

Figure S8. The mass loading versus current density measured at a constant overpotential of 200 mV for the QAMN and CMN electrodes.

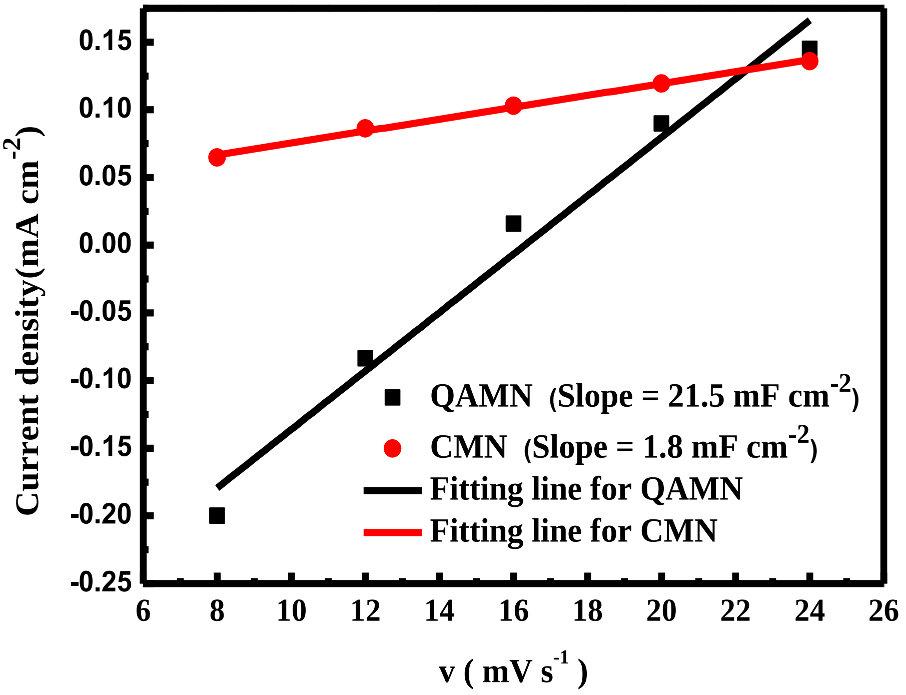

Figure S9. Double-layer capacitance measurement of the QAMN and CMN electrodes in 0.1 M KOH solution for comparing the electrochemical active surface area. Cyclic voltammograms of these two electrocatalysts were measured in a non-Faradaic region at different scan rates. The current density measured at 0.66 V vs. RHE was plotted as a function of scan rate.

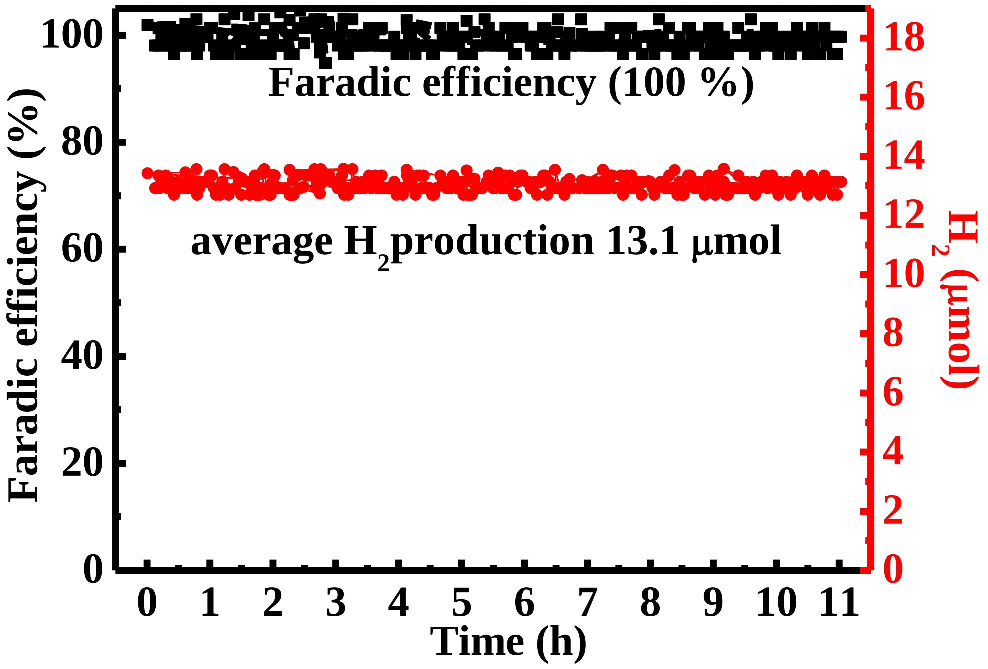

Figure S10. The Faradaic efficiency and the H_2 production of the sample QAMN with mass loading of 0.26 mg cm^{-2} for alkaline HER. The fluctuation of the Faradaic efficiency is attributed to the adsorption and desorption of the H_2 bubbles on the electrode surface.

Table S1. Previous metallic nickel electrocatalysts for alkaline HER at 25 ^{o}C.

Catalysts	loading (mg cm^{-2})	electrolyte	TOF (s^{-1})	J_0 (mA cm^{-2})	η at J=10 mA cm^{-2} (mV)	Reference and onset potential
**QAMN**	**0.26**	**0.1M KOH**	**0.838 (200 mV)**	**0.75**	**240**	**this work (0 mV)**
Ni dendrite	/	6 M KOH	/	0.047	382	[1]
Ni particle	/	6 M KOH	/	0.029	412	[1]
Ni film	/	6 M KOH	/	0.020	452	[1]
Ni foil	/	6 M KOH		0.015	532	[1]
Ni	/	1MKOH	/	0.0026	379 (J=1 mA cm^{-2})	[2]
Macroporous Ni	/	1MKOH	/	0.0002	348 (J=100 mA cm^{-2})	[3]
Crystalline Ni	/	1MKOH	/	/	250	[4]
Amorphous Ni alloys		1MKOH	/	/	450	[4]

TOF calculations[5]: Measurement of mass−specific catalytic activities enables an estimation of the activity of CMN and QAMN on a per-surface-atom basis, given a series of approximations regarding particles composition and morphology. For the CMN catalyst, the total surface area of 0.1 mg of sheets, 100 nm sheet diameter with a density of 5.36 g mL^{-1} (based on the weighted−average density of Ni:Mo) is ~22.4 cm^2. It implies that the roughness factor, γ, for a 0.1 mg cm^{-2} sample is ~22.4. Assuming that the sheets surfaces exhibit the weighted-average lattice constants of their bulk Ni, Mo components, 0.1 mg of material contains 1.68 μmol of surface atoms. Hence for films with low mass loading, the turnover frequencies can be estimated as 0.033 s^{-1} (Excel CMN) at η = 200 mV. For the QAMN catalyst, the total surface area of 0.1 mg of spherical, 25 nm diameter particles with a density of 9.94 g mL^{-1} (based on the weighted-average density of Ni:Mo:W) is ~23.3 cm^2. It implies that the roughness factor, γ, for a 0.1 mg cm^{-2} sample is ~23.3. Assuming that the nanoparticle surfaces exhibit the weighted-average lattice constants of their bulk Ni, Mo, W components, 0.1 mg of material contains 1.04 μmol of surface atoms. Hence for films with low mass loading, the turnover frequencies can be estimated to be 0.838 s^{-1} at η = 200 mV (Excel QAMN).

More details see the Supplementary Methods and Excel.

γ: The roughness factor

Table S2. Surface atoms calculation parameters at η=200 mV (more details in Excel)

Sample ID	Averaged density (gmL^{-1})	Molar volume ($mLmol^{-1}$)	Surface area in 0.1 mg (cm^2)	Surface atoms in 0.1 mg (mol)	Size in diameter (nm)	TOF (s^{-1})	Power law equation
CMN	5.36	11.09	22.4	1.68	100	0.033	$y=8.46*x^{1.698}$
QAMN	9.94	6.14	23.3	1.04	25	0.838	$y=31.855*x^{0.665}$

References:

[1] S. H. Ahn, S. J. Hwang, S. J. Yoo, I. Choi, H. J. Kim, J. H. Jang, S. W. Nam, T. H. Lim, T. Lim and S. K. Kim, J. Kim, *J. Mater. Chem*. **2012**, *22*, 15153-15159.

[2] E. Navarro-Flores, Z. W. ChongSasha and S. Omanovic, *J. Mol. Catal. A- Chem*, **2005**, *226*, 179-197.

[3] C. González-Buch, I. Herraiz-Cardona, E. M. Ortega, S. Mestreand, V. Pérez-Herranz, *Int. J. Hydrogen Energy* **2016**, *41*, 764-772.

[4] D. W. Kirk, S. J. Thorpe and H. Suzuki, *Int. J. Hydrogen Energy* **1997**, *22*, 493-500.

[5] J. R. McKone, B. F. Sadtler, C. A. Werlang, N. S. Lewis and H. B. Gray, *ACS Catal.* **2013**, *3*, 166-169.
